# A comparative study of using scorpion antivenom versus scorpion antivenom and prazosin drug for scorpion stings management in Sohag University Hospitals

**DOI:** 10.1186/s40360-025-00854-x

**Published:** 2025-02-14

**Authors:** Meray Medhat Shokry Zaghary, Mai Mostafa Abd ElKader

**Affiliations:** Department of Forensic Medicine and Clinical Toxicology, Faculty of Medicine, Sohag University, Sohag, Egypt

**Keywords:** Scorpion envenomation, Prazosin, Antivenom, Efficacy

## Abstract

**Background:**

Scorpion envenomation is a worldwide problem, especially in tropical and subtropical areas like Egypt. Scorpion envenomation is responsible for the high mortality rate all over the world, which makes much research carried out to see the efficacy of other drugs as supportive treatment in addition to antivenom.

**Aim:**

To see the efficacy of prazosin drug with scorpion antivenom compared to antivenom alone.

**Methods:**

It is a prospective randomized comparative study between two groups from February 2023 to July 2024. Each group is 50 cases to compare the efficacy of prazosin with antivenom (group 1) and the antivenom alone (group 2) in scorpion stings cases.

**Results:**

The median age of the 100 cases was 7 years old. 54% of the study population were males. 46% of cases were females. Most of the cases were from rural areas. There was no significant difference in age, sex, and the patients’ residence between the two groups. There was no significant difference between the 2 groups regarding the duration of stay in the hospital till mortality or discharge, with a median of 3 days in each group. However, there was a significant difference in the antivenom received in each case between the 2 groups, with a median of 6 vials in group 1 and 9 vials in group 2. Mortality and complications were observed to occur more in group 2 than in group 1 despite the insignificant p values. The study’s mortality rate was 14%, 4/50 (8%) in group 1 and 10/50 (20%) in group 2. The number of antivenoms significantly increased with an increase in duration of stay, heart rate, respiratory rate, troponin level, and mortality outcome. While the number of antivenoms significantly decreased with the rise in systolic and diastolic blood pressure.

**Conclusion and recommendations:**

: The study concluded that prazosin can be added to antivenom to increase its efficacy and decrease the number of needed antivenoms. Prazosin is a safe drug when used with precautions to avoid the first-dose phenomenon. Prazosin decreases complications and mortality when added to antivenom, but not significantly. The study recommends using prazosin with precautions for all manifested scorpion cases with antivenoms to increase the efficacy of the antivenom and treat the adrenergic manifestation.

## Introduction

Scorpion envenomation (SE) is a common health issue, particularly in tropical and subtropical nations like Upper Egypt, which is thought to be a significant risk factor for morbidity and mortality, especially in children [1].

The World Health Organization (WHO) estimates that each year, between 1.2 and 1.5 million people get a scorpion sting, and between them, about 3,000 and 5,000 people die in the world due to scorpion envenomation [2].

There are about 2500 scorpion species worldwide, and about 58 subspecies are dangerous for humans with medical importance. The Scorpion (Buthidae) families contain the most lethal species [3]. An Egyptian study revealed that Leiurus quinquestriatus scorpion and Androctonus crassicauda are the most common cause of scorpion stings in southern regions. These two species belong to the Buthidae family and are mainly found in North Africa, the Middle East, and Asia [4].

Several factors can affect clinical manifestations of scorpion sting, including the dose of venom, the age of the affected child, the season of the sting, and most importantly, the time lapse between the sting and hospitalization. Also, the sex of the child, pre-hospitalization first aid, and the interval between sting and prazosin administration have been suspected of playing an essential role in the development of complications and subsequently adverse outcomes [5].

There have been trials with various regimens, including antivenom, vasodilators, inotropic support, and metabolic rectifiers such as insulin and L-carnitine. The only treatment for the etiology is immunotherapy. Early administration prevents many complications and improves outcomes. Additionally, Symptomatic treatment is still necessary to support immunotherapy, particularly in delayed hospital arrival cases. Regarding local resources and limitations, a combination of both strategies should be considered [6].

In recent years, prazosin, an alpha-1 blocker, has been used to treat severe scorpion stings and is thought to be the principal treatment of the autonomic scorpion envenomation storm [7]. Scorpion-envenomating cases with arrhythmia or hypertension can be treated with prazosin alone or with antivenom. In cases with acute pulmonary edema, heart failure, or shock, it was advisable to use dobutamine instead of prazosin [8]. The efficacy of prazosin, when added to antivenom to prevent complications, is still an interesting point for research.

## Aim of the study

The objective is to see the efficacy of prazosin drug with scorpion antivenom compared to antivenom alone on mortality, complications, number of antivenom vials needed, and duration of hospitalization.

## Patients & methods

### Type of study

It is a prospective randomized comparative study carried out during the period from February 2023 to July 2024.

### Randomization

Single-anonymized randomization was done by code number from 1 to 100. Randomization was done by a random website generator, which was https://www.random.org/. The random code number was distributed in an Excel sheet before starting the trial sequentially into scorpion antivenom plus prazosin (group 1 or experimental group) and scorpion antivenom (group 2 or control group). Each number was put in an opaque-sealed envelope for every patient to ensure concealed treatment allocation. Cases with warm shock or severe shock were put in group 2 and take code in group (2) with patient concealed treatment.

### Sample size

Sample size calculation by OpenEpi program, version 3 open source calculator -SSCC using randomized clinical trials study equation when we depend on the previous results of Shoreit et al. [9]. When we assumed that mortality in cases would take antivenom only about 21%, in comparison, mortality in the group will take the combination of antivenom and prazosin about 3%, and control number to case number were equal, within an error probability of 0.05 and 80% power on 2- tailed test (type 1 error). A type 1 error, a false positive result, occurs if the researcher rejects a true null hypothesis. A type 2 error, a false negative result, occurs if the researcher fails to reject a false null hypothesis. Calculating a reasonable sample size was needed to avoid the two types of errors [10].

It was calculated that we needed about 50 samples in each group.

***Inclusion criteria***:

- All patients admitted to Sohag University Hospitals with a history of scorpion stings from February 2023 to July 2024 after accepting informed consent from the case itself or the first-degree relative if the case is underage or in a coma. The cases included were manifested with any of the typical scorpion manifestations like pain, vomiting, priapism, shock or hypertension, salivation, arrhythmia, toxic myocarditis, or pulmonary edema.

***Exclusion criteria***:

All other envenomation cases or poisoning were excluded. All cases without clinical manifestations were excluded.

### Tools of the study

1. Dose and administration of prazosin: 30 µg/kg/dose of prazosin was delivered orally every 6 h, 4 doses, and not exceeding 1 mg per dose. Prazosin had been delivered using a nasogastric tube while securing the patient’s airway in the event of vomiting or unconsciousness. Every 30 min for the first three hours, every hour for the following six, and then every four hours until improvement, blood pressure, pulse rate, respiration rate, and oxygen saturation were measured. If patients were presented with pain as the only symptom, prophylaxis would not be needed. The patient was kept in a lying position for around 3 h to avoid the first-dose phenomenon [9].
2. Dose of antivenom 1 ml intramuscular and/ or 1 to 5 polyvalent anti-scorpion vials produced by the vaccine & serum institution (VACSERA) in Egypt according to the severity of the case on 200 to 500 ml glucose 5% infusion and then the patient assessed clinically to repeat the infusion dose after 4 to 6 h [6].
3. A prepared sheet containing:
  The demographic characteristics of each patient.
  Vital signs and degree of coma at the time of admission of the patient.
  First aid management as resuscitation.
  Supportive management was recorded for the patients during admission.
  Cardiac enzyme measurements, hospital stay duration, and patient outcomes in each group were recorded.

### Statistical analysis

Data were fed to the computer and analyzed using IBM SPSS software package version 26.0. **The** Qualitative data were described using numbers and percentages. The Kolmogorov-Smirnov test was used to verify the normality of distribution. Quantitative data were described using range (minimum and maximum), median, and interquartile range (IQR). The significance of the obtained results was judged at the 5% level.

**The tests used were**

1. **Chi-square test**: for qualitative data
2. **Mann Whitney test**: to compare the difference between 2 independent non-parametric quantitative data.
3. **Spearman correlation**: to correlate between essential parameters in the study.

### Ethical conditions

Ethical approval was obtained from the Medical Research Ethics Committee of the Faculty of Medicine—Sohag University On 8/2/2023 under IRB registration number Soh-Med-23-02-24. All patients or first-degree relatives had signed informed consent before participating in the study and could refuse or accept this participation. The Helsinki Declaration and its following amendments set forth the relevant ethical norms, directives, and laws, which were followed in executing all the study’s techniques.

ClinicalTrials.gov. Protocol Registration and Results System (PRS) Receipt Release Date: February 29, 2024 ClinicalTrials.gov ID: NCT06287905.

## Results

**Fig. 1:**
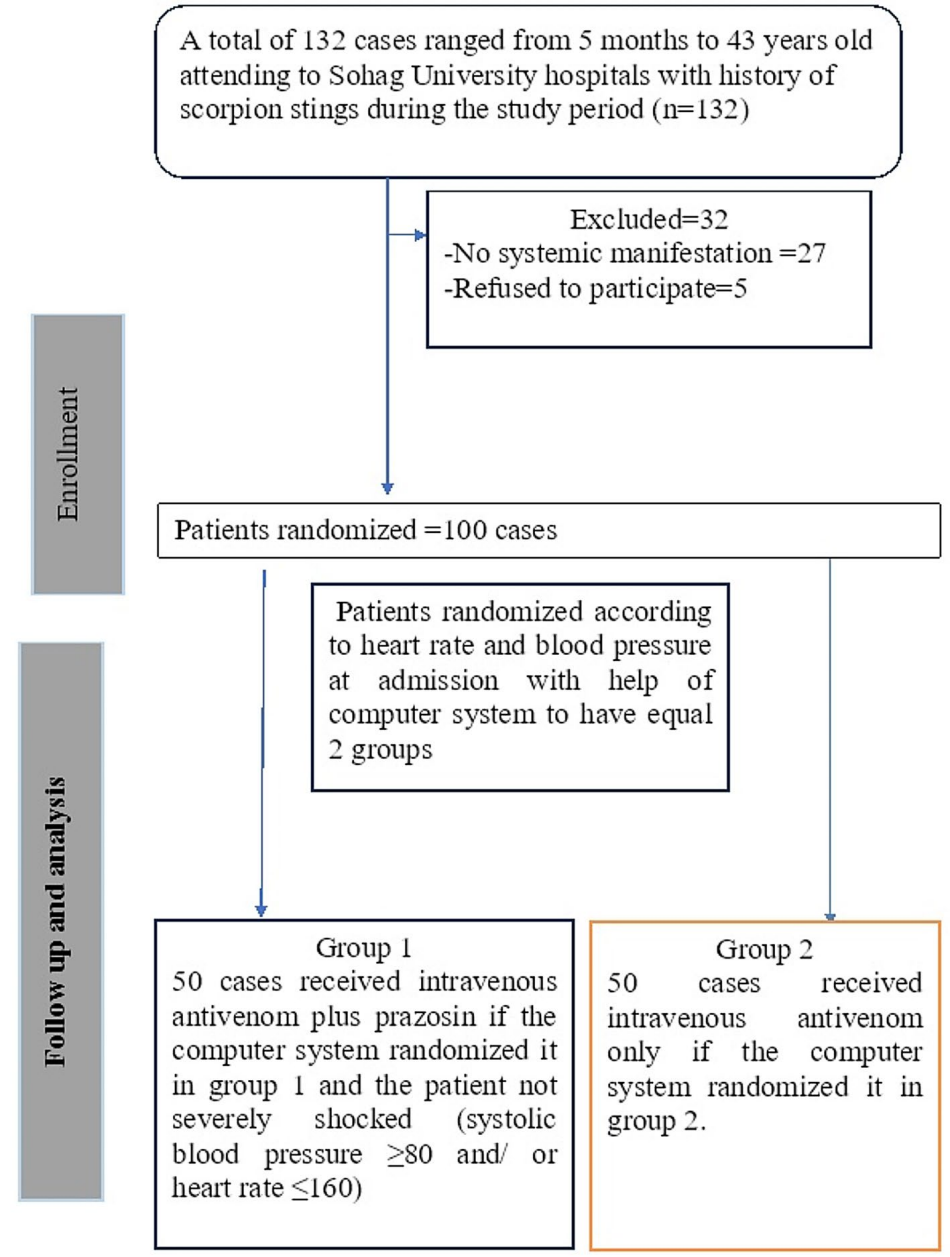
Flow chart of the included cases in the study

A total of 132 cases were initially included in the study. Thirty-two cases were excluded; twenty-seven were without clinical manifestation, and five were refused participation (Fig. 1).

The median age of all cases was 7 years old. 54% of the study population were males. 46% of cases were females. Most of the cases were from rural areas. There was no significant difference in age, sex, and the patients’ residence between the two groups.

There was no significant difference between the 2 groups regarding Abroug’s grading, PSS, GCS, respiratory rate, troponin level, presence of vomiting or priapism, fever, pain and shock, and site of admission after coming to the hospital as P values were > 0.5.

Both groups had a significant difference regarding heart rate and blood pressure, as group 2 had a median heart rate of 130 beats per minute, significantly more than group 1 = 120 beats. A median of systolic and diastolic blood pressure significantly decreased in group 2 than in group 1, as cases with severe shock were excluded from receiving prazosin (Table 1).

**Table 1:** Demographic and clinical data of the study population (*n* = 100)

Parameters		Group 1 (*N* = 50) 100%	Group 2 (*N* = 50) 100%	Total (*N* = 100)	*P* value
Age (years)	Median (IQR)	7(4-11.2)	7(4-11.2)	7(4-11.2)	
	Range	0.5–39 years	0.5–43 years	0.5–43	0.9^a^
Gender	Male	23(46%)	31(62%)	54 (54%)	0.1^b^
	Female	27(54%)	19 (38%)	46 (46%)	
Residence	Urban	6(12%)	3 (6%)	9(9%)	0.29^b^
	Rural	44(88%)	47 (94%)	91(91%)	
Abroug’s grading	1	6(12%)	4(8%)	10 (10%)	0.18^b^
	2	19(38%)	12(24%)	31(31%)	
	3	25 (50%)	34(68%)	59(59%)	
Poisoning severity score at presentation	1	5(10%)	3(6%)	8 (8%)	0.5^b^
	2	19(38%)	14(28%)	33(33%)	
	3	22 (44%)	26 (52%)	48(48%)	
	4	4 (8)	7(14%)	11(11%)	
GCS at presentation	Median (IQR)	14(12–15)	13(8–15)	14(9–15)	0.1^a^
	Range	3–15	3–15	3–15	
Systolic pressure at presentation	Median (IQR)	100(83.7-112.5)	90(80–100)	100(80–110)	0.005*^a^
	Range	80–150	55–120	50–150	
Diastolic pressure at presentation	Median (IQR)	70(50-72.5)	60(50–60)	60(50–70)	0.001*^a^
	Range	40–100	20–80	20–100	
Heart rate at presentation	Median (IQR)	120(100–140)	130(120-152.5)	130(110–140)	0.001*^a^
	Range	60–180	90–235	60–235	
Respiratory rate at presentation	Median (IQR)	22(20-26.2)	25(19.7–30)	23(20–30)	0.4^a^
	Range	7–80	7–50	7–80	
Troponin level	Median (IQR)	1(0.2-3)	2(0.1–7.2)	1.8(0.1–5.3)	0.2^a^
	Range	0–25	0-3000	0-3000	
Clinical manifestations	Vomiting	41/50(82%)	32/50(64%)	73/100(73%)	0.07
	Pain	25/50 (50%)	21/50(42%)	46/50 (46%)	0.5
	Priapism	8/50(16%)	7/50(14%)	15/100(15%)	0.77
	Fever	9/50 (18%)	5/50(10%)	14/100(14%)	0.3
	Shock	3/50(6%)	9/50(18%)	12/100(12%)	0.1
Site of admission	Inpatient ICU	28(56%) 22(44%)	20(40%) 30(60%)	48(48%) 52(52%)	0.1^b^

^a^By Mann Whitney test ^b^By Chi-square test *P value < 0.05 is considered significant

There was no significant difference between the 2 groups regarding the duration of stay in the hospital till mortality or discharge, with a median of 3 days in each group. However, there was a significant difference in the antivenom received in each case between the 2 groups, with a median of 6 vials in group 1 and 9 vials in group 2 (Table 2).

**Table 2:** Comparison of the outcome of the 2 groups of the study (*n* = 100)

Parameters		Group 1 (*N* = 50) 100%	Group 2 (*N* = 50) 100%	Total (*N* = 100)	*P* value
Duration of stay(days)	Median (IQR)	3(2–5)	3(2–5)	3(2–5)	0.2^a^
	Range	1–15 days	1–15 days	1–15	
Number of antivenom	Median (IQR)	6(3–12)	9(5–14)	6.5(3.3–12)	0.04*^a^
	Range	1–25	1–26	1–26	
Outcome	Rapid recovery	35(70%)	30(60%)	65 (65%)	0.2^b^
	Complicated recovery	11(22%)	10(20%)	21 (21%)	
	Mortality	4(8%)	10(20%)	14(14%)	
Survival	Recovered	46(92%)	40(80%)	86(86%)	0.08^b^
	Died	4(8%)	10 (20%)	14(14%)	

^a^By Mann Whitney test ^b^By Chi-square test *P value < 0.05 is considered significant

**Fig. 2:**
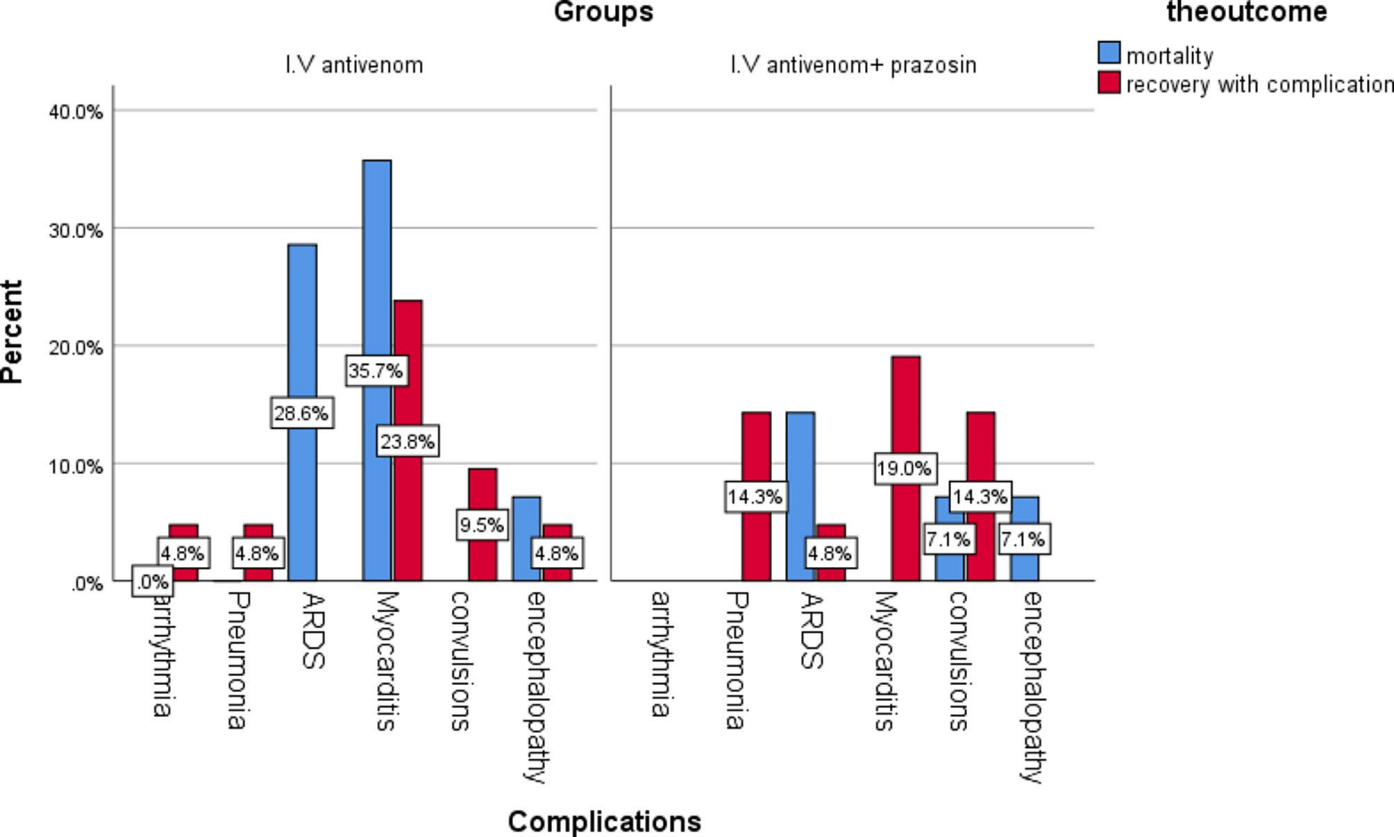
Bar chart of distribution of complications according to outcome in the 2 groups

Mortality and complications were observed to occur more in group 2 than in group 1 despite the p values being insignificant. The study’s mortality rate was 14%, 4/50 (8%) in group 1 and 10/50 (20%) in group 2 (Table 2). Complications incidence was 35/100 (35%), 15/50 in group 1, and 20/50 in group 2—(Table 3). Pulmonary oedema (6 cases), toxic myocarditis (5 cases), toxic encephalopathy (2 cases), and refractory convulsions (1 case) were the complications associated with death, respectively (Fig. 2).

**Table 3:** Comparison of occurrence of complications in the 2 groups of the study (*n* = 35)

Parameters		Group 1 (*N* = 15)100%	Group 2 (*N* = 20) 100%	Total (*N* = 100)	*P* value
Complications	Arrhythmia (SVT)	0(0%)	1(5%)	1 (2.9%)	0.14
	Pneumonia	3(20%)	1(5%)	4(11.4%)	
	Pulmonary edema	3 (20%)	4(20%)	7(20%)	
	Myocarditis	4 (26.7%)	10(50%)	14(40%)	
	Convulsions	4(26.7%)	2(10%)	6(17.1%)	
	Encephalopathy	1(6.7%)	2(10%)	3(8.6%)	

By chi-square test

SVT: Supraventricular tachycardia

There was a positive, significant mild correlation between increasing antivenom and a group of therapy (1 with prazosin and 2 without). Heart rate was negatively correlated with group therapy, while systolic and diastolic blood pressure were positively correlated with treatment (1 with prazosin and 2 without).

The number of antivenoms significantly increased with an increase in duration of stay, heart rate, respiratory rate, troponin level, and mortality rate. While the number of antivenoms significantly decreased with the rise in systolic and diastolic blood pressure.

The duration of stay increased significantly with the increasing respiratory rate and decreased mortality. (Table 4)

**Table 4:**
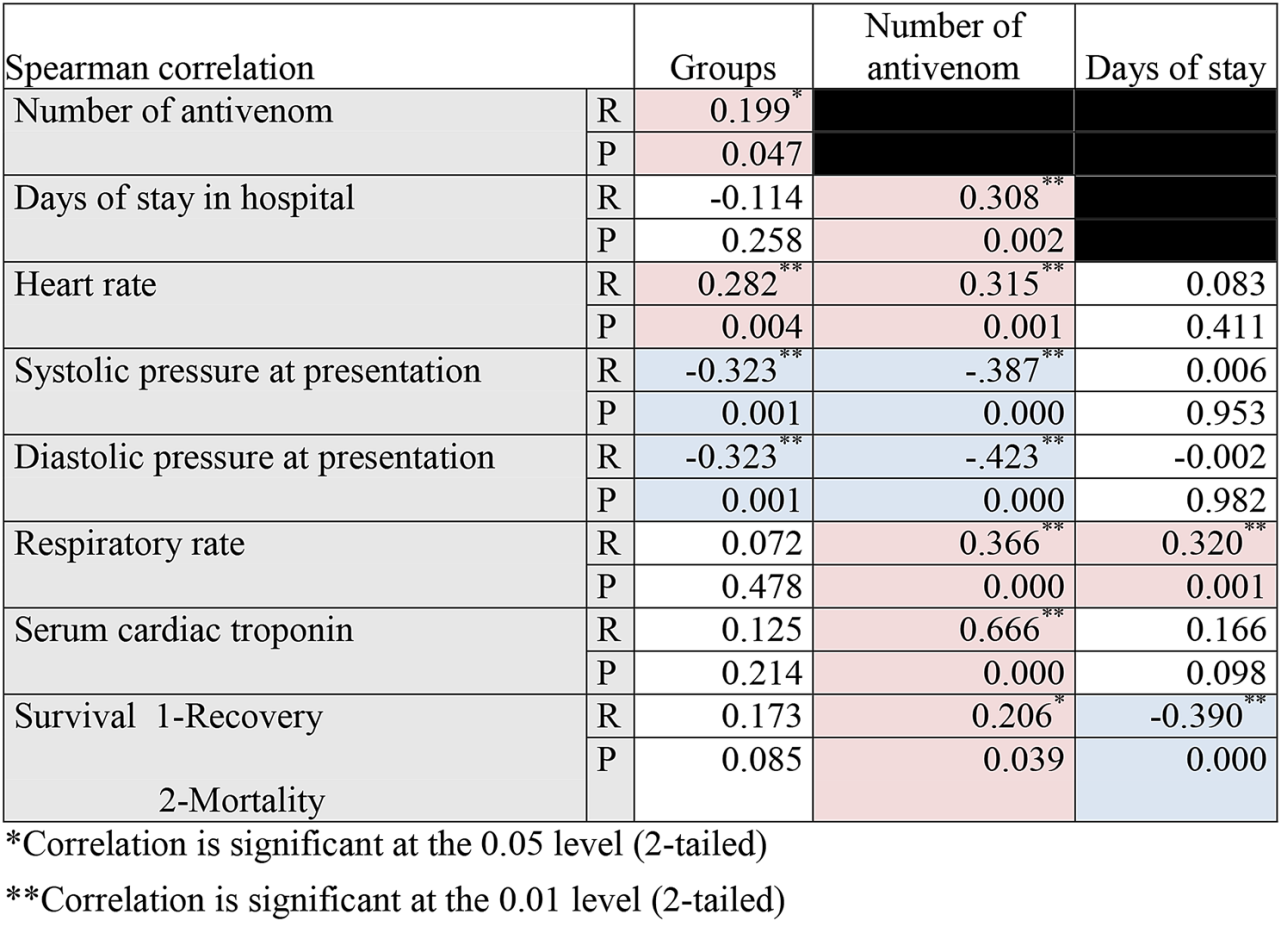
Spearman correlation of essential parameters in the study number = 100

## Discussion

Scorpion envenomation is a life-threatening public health hazard in tropical and sub-tropical regions of the world. Prazosin is considered a physiological and pharmacological antidote to scorpion venom action. It antagonizes the after-effects of venom-liberated catecholamines. Early administration of prazosin helps arrest the development of severe systemic features, thereby preventing mortality. Compared to the pre-prazosin era, the mortality rate due to scorpion envenomation has reduced from 30 to 1% after routine use of prazosin. Currently, the simultaneous use of prazosin and scorpion antivenom is considered the mainstay of treatment of scorpion envenomation [11].

The median age of all cases in the study was 7 years old, and this was similar to a study conducted by Mourad et al. [12]; the highest percentage of patients who represented scorpion stings (29.4%) was in the age group less than 10 years. Also, Ismail et al. [13] revealed that the mean age of the studied cases was 9.48 ± 0.5 years. This age median was close to Meki et al. [14], as the mean age of their patients was 7.3 ± 0.72 years. On the contrary, Yuvaraja et al. [15] stated that the mean age of the patients was 34.8 ± 13.6 years. Children are more susceptible to scorpion envenomation due to their inquisitive nature and risk-taking behavior [5].

There was male predominance as 54% of the study population was males, while 46% of cases were females, following Prasad et al. [16], who stated that males (65%) were predominantly affected. Also, in other studies by Alhamoud et al. [17], Firoozfar et al. [2], and Laxmanan & Vengadakrishnan [18], the majority of the patients were males. In a study by Chippaux et al. [19], there was no difference between the incidence of stings in males and females. The difference in the percentages between males and females was attributed to the more outdoor time spent by males than females, which put them at more risk for scorpion stings [1].

Most cases were from rural areas, which agrees with other studies [4, 9].

Regarding Abroug’s grading, most cases were in grade 3, while according to PSS, most were in grade 3, followed by grade 2, with no significant difference between the 2 studied groups. The distribution of grades of envenomation agreed with Meki et al. [14] as all of their patients had severe envenomation (grade III of Abroug’s grading) and disagreed with Kannan et al. [13] and Ismail et al. [20]. This difference can be explained by the different times of hospital arrival after stings, which affect the appearance of clinical manifestation.

In the present study, there was a significant difference between both groups regarding heart rate and blood pressure as group 2 had a median heart rate of 130, which is significantly increased than the median of group 1 = 120. The median of systolic and diastolic blood pressure was 100 and 60, respectively, with a significant decrease in group 2 than group 1. Heart rate was negatively correlated with group therapy, while systolic and diastolic blood pressure was positively correlated with treatment (1 with prazosin and 2 without). Abd El-Aziz et al. [5] stated that the mean systolic and diastolic Bp were (116.11 ± 14.33 and 73.89 ± 5.58, respectively). A study by Shoreit et al. [9] revealed that adding prazosin to antivenom improved tachycardia and induced earlier clinical recovery than in cases treated with conventional therapy only. These results agreed with other studies, such as by Khalaf et al. [21], who showed that heart rate normalization occurred after adding prazosin. Hypotension can occur within 1–2 h after the sting due to fluid loss and within 4–48 h due to left ventricular dysfunction [22]. 

Regarding clinical manifestations, vomiting was the most common presentation in 73% of cases, followed by pain in 46% of cases, while priapism, fever, and shock were in 15%, 14%, and 12% of cases, respectively, with no significant difference between the 2 studied groups. These results agreed with Prasad et al. [16], who reported that pain (62.5%) was the most common local manifestation, vomiting (32.5%) and priapism (50%). In a study by Shoreit et al. [9], all patients had pain at the site of sting in 60 (100%) cases, vomiting in 58 (96.7%) cases, and priapism in 10 (16.7%) cases with significant difference between cases who treated with conventional therapy only and those who treated with conventional treatment and prazosin.

Troponin level increased in envenomated cases with a median level of 1.8, closer to Abdel Baseer et al. [4], but there was no significant difference between the 2 studied groups.

In the present study, 48% of cases were admitted to inpatient. In comparison, 52% of cases needed ICU admission, which agreed with Abdel Baseer et al. [4], as sixty-four cases suffered systemic organic complications and needed ICU admission.

Regarding the duration of stay in the hospital, there was no significant difference between the 2 groups, with a median of 3 days in each group. El-Aal et al. [23] reported that the mean duration of hospitalization was 1.720 ± 0.9802. In contrast, Shoreit et al. [9] stated that the duration of hospital stay was less in the group that received prazosin than in the group that received antivenom only with earlier recovery, which agreed with Arivoli & Ganesh [24], who found that 82.3% stayed for < 3 days and 17.6% stayed for > 3–5 days with the use of prazosin.

In the present study, there was a significant difference in the antivenom received in each case between the 2 groups, with a median of 6 vials in group 1 and 9 vials in group 2. There was a positive, significant mild correlation between increasing antivenom and a group of therapy (1 with prazosin and 2 without). This result agrees with El-Aal et al. [23], who found that the mean number of scorpion antivenom was 6.5.

Regarding the outcome, survivors represent 86%, while mortality represents 14%, 4/50 (8%) in groups 1 and 10/50 (20%). Mortality and complications were observed to occur more in group 2 than in group 1 despite the insignificant p values. The percentage of the mortality rate was closer to El-Asheer et al. [6] and Ahmed et al. [7]. In a study by Shoreit et al. [9], mortality was 8.3%, with a difference between the group treated with prazosin (3.3%) and the group that didn’t receive prazosin (13.3%). Also, Bahloul et al. [25] reported that the mortality rate was reduced to 1% compared to a 30% mortality rate in the pre-prazosin period.

The incidence of complications in the study was 35/100 (35%), with 15/50 in group 1 and 20/50 in group 2. Pulmonary oedema was in 6 cases, followed by toxic myocarditis (5 cases), toxic encephalopathy (2 cases), and refractory convulsions (1 case) were the complications associated with death, respectively. These results go in harmony with a study by Abdel Baseer et al. [4] in which myocarditis was detected in 28 (44%) cases and pulmonary edema was detected in 13 (20.3%) cases, convulsions 11%, shock 48.5%, and sinus tachycardia was the most common electrocardiographic changes detected (21cases). Also, in Shoreit et al. [9], pulmonary edema occurred in six (10%) cases and myocarditis in 11 (18.3%) cases, with no significant difference between the two studied groups. The percentage of each complication was close to other studies by Yuvaraja et al. [15] and El-Aal et al. [23].

The number of scorpion antivenoms significantly increased with the duration of stay, heart rate, respiratory rate, troponin level, and mortality rate. In contrast, the number of antivenoms decreased considerably with increased systolic and diastolic blood pressure. El-Aal et al. [23] mentioned that the severely manifested group with high troponin levels needed more antivenom vials and more duration of hospitalization.

### Limitations of the study

There are limitations in this trial that need to be recognized. It was a single-anonymized trial with an inherent risk of bias. Blood pressure and pulse were measured at the time of arrival in the emergency room, and if the patient accepted the consent, he was included in the study. We didn’t make stratified randomization since using heart rate and blood pressure as covariables.

The study included patients with a history of stung by scorpion and presented with typical scorpion envenomation manifestations. Few patients can remember the size, color, and type of scorpion that stung them. The type of species that caused the stings of the people in the study was not recorded.

## Conclusion and recommendations

The study concluded that prazosin can be added to antivenom to help the recovery of scorpion cases and decrease the number of needed antivenoms. Prazosin is a safe drug when used with precautions to avoid the first-dose phenomenon. Prazosin decreases complications and mortality when added to antivenom, but not significantly. The study recommends using prazosin with precautions for all manifested scorpion cases with antivenoms to increase the good prognosis of symptomatized scorpion cases and treat the adrenergic manifestations.

## Data Availability

The raw data with the corresponding author that can be provided by direct request.
